# Notices

**Published:** 1863-05

**Authors:** 


### NOTICES.

The American Tract Society, 28 Cornhill, Boston, and 13 Bible House, Astor Place, New-York, has issued the *Wicket Gate*, beautiful in manner and sweet in substance, being "Short Narratives of the turning of sinners to God, with words of counsel and warning;" also *The Senses*, with numerous illustrations of sight, taste, touch, hearing, smell, etc., the motto being from Bayne, "Christ exalted our whole conception of nature, by habitually associating it with the spiritual instruction of man;" also *The Way to be Happy, or Willie the Gardener-Boy*, by Catherine D. Bell, who has also written a deeply interesting and instructive narrative under the title of *The Two Ways*. Mr. J. G. Broughton, at the Bible House Branch, has every variety of publications for the instruction and comfort of old and young, and we counsel our readers to go there the very first place when they come to the city, and get their best money's worth before it has slipped away for less valuable things than the Boston Tract Society's publications.

Exchanges, whom it may concern, take notice that the Editor of Hall's Journal of Health feels greatly obliged to them for the partiality with which they regard his writings. We constantly feel when we see our pieces copied, "I must try and make them more worthy of being transferred to the pages of other periodicals," as we see our defects a great deal sooner and more distinctly than others possibly can do, for we were born a cricket, in other words a critic, but we have a gift very uncommon, and that is when a criticism is adverse, to examine its correctness with perfect good-nature, and then to profit by it, however belligerently or spitefully the criticisms were made, because we know that the gift of kindly criticism is a divine emanation.

In one thing we would like our exchanges to be a little more on their guard as to a small matter of right and wrong. They are sometimes careless in giving us credit for our good things; and inasmuch as the fact of exchanging is often the merest act of courtesy, without any other *quid pro quo* than the mere weight of the paper at six cents a pound, we think we ought to be helped along a little. The *Transcript* of Philadelphia lately copied three or four pages bodily, and "left no sign" as to where it came from. The *Saturday Evening Post*, a veteran paper of whose pages there are many, over fifty years, who have pleasant memories, will take an article, cut it up into giblets, and scatter it all over its pages. See our April issue about Baldness, etc. The *New-York Traveller*, in which friend Ropes manages to twine in so many good pieces, takes our Health Tract on "Vermin Riddance," heads it "Things that Bite and Sting," and by giving no credit makes his readers believe that he believes we write so nearly as well as he does, that they can't tell the difference; and in the same issue he gives more than two other pages of matter from our Journal, which, though not our own, and given as an illustration of our idea what true mental recreation was, would have afforded an easy opportunity of referring to us in a friendly way. But that is not the way that the *Home Journal* of Morris and Willis, (grown increasingly valuable and instructive of late,)

and the *Scientific American* of this city do things. See how openly and above-board the latter prefaces three or four columns of extracts from our April No. on Housekeeping, Whitewashes, Who are to Fight, etc. But it is a way which Munn & Co. have, open, manly, honorable, extending alike to the whole range of their business matters, whether as to their paper or their admirable and prompt manner of obtaining patents for the ingenious and the gifted, as thousands of inventors will testify. "There is no periodical on the list of our exchanges that we welcome more warmly than Hall's Journal of Health. Our readers are indebted to it for many interesting and valuable suggestions regarding their moral and physical health, and also matters relating to domestic economy, which we from time to time extract from its pages. The articles are always well written and convey the author's ideas lucidly and forcibly. We commend the above-named periodical to all persons desiring to obtain useful knowledge at a very low rate. Dr. Hall is doing a lasting good by disseminating valuable information in a proper form."

Practical Inference—It is such a nice thing to do right, to do things "on the square," as poor Dan Marble said he had tried to do, when he was just dying of cholera; that we wonder every body don't do it, just for the love of it; besides, it's most sure to prove profitable pecuniarily sooner or later; and in this case sooner, for we were so pleased with the candid spirit of the writer, we said to a friend, "Munn & Co.'s a good fellow, give him ten dollars' worth of advertising," and he did! For be it known, that persons not only come to the Editor of the Journal of Health to get advice as to how they are to get well or keep well, but as to who and when they ought to marry, what kind of business they should go into, how they are to get employment, but also what is the best paper to advertise in. We have generally said, if you want to get the ear of the intelligent and thinking, advertise in the *Scientific American*. If you want to reach the rich and refined, those who have taste and cultivation, go to the *Home Journal;* if you want to reach the solidly good families who are religious from principle, take *The Presbyterian* of Philadelphia or the *Christian Intelligencer* of New-York City. If you want to reach active, live, working Christians, spread yourself out in the columns of the *Evangelist.* If you desire to secure the attention of every-thing-arians whose mental and pecuniary acquisitions are generally on a par with —— "Nary a red," then take the *New-York Weekly*—ahem! how do you spell it? If our exchanges are not more particular in giving us proper credit, the result will be, that whenever the public sees any thing in a newspaper, really good, practical, succinct and plain, without a name, they will take it for granted that it is from Hall's Journal of Health.

Country Milk, pure and fresh, is a matter literally of vital importance to city families, especially in summer, and where there are young children; and every parent should make it an object of specific personal inquiry and investigation, as to where pure, rich, fresh milk can be had; this is done with the utmost certainty, promptness, and regularity under the auspices of G. W. Canfield, Esq., at 146 Tenth street, near Broadway, and at the lowest price, as we personally know, after a four years' most satisfactory experience.

